# Morbidity and Related Factors among Elderly People: A Cross Sectional Study Exploring Gender Differences

**Published:** 2020-02

**Authors:** Abdelhafid BENKSIM, Rachid AIT-ADDI, Noureddine ELKHOUDRI, Mohamed AMINE, Mohamed CHERKAOUI

**Affiliations:** 1. Laboratory of Human Ecology, Semlalia School, Marrakesh, Morocco; 2. High Institute of Nursing and Technical Health (ISPITS), Marrakesh, Morocco; 3. Institute of Health Sciences, University Hassan-1, Settat, Morocco; 4. Faculty of Medicine and Pharmacy, Marrakesh, Morocco

## Dear Editor-in-Chief

Currently, population aging presents a new challenge for all the countries in the worldwide. It is a natural and inevitable process, always associated with physiological and biological decline (1). Longer life is related to chronic conditions and disabilities in advanced ages, which affected the quality of life and pose a challenge to families and communities (1). Elderly women are more likely to suffer from lower socio-economic and chronic morbidities that make them more dependent on their daily lives. Our survey was conducted to distinguish the gender differences and the associated factors in elderly in the Marrakech City, Morocco.

A group of 368 older persons aged 60 yr and above were successfully included by accidental non-probabilistic sampling through face-to-face interview.

Study protocol was explained and informed consent was obtained. Exclusion criteria were elders with dementia or speech impairments. Pearson's chi-square test is used for analysing data provided by SPSS software (Chicago, IL, USA). *P-*value <0.05 was statistically significant.

Table 1 sets the socio-demographic and clinical characteristics of participants in this study group. In this context, 53.8% of men are ranged between 70 to 98 yr old. They are more likely to live longer with a partner for the rest of their lives (58%). This finding may be due to remarriage trends observed among men and also to complications in pregnancy or childbirth in developing countries. In contrast, females universally experience longer life expectancy in developed countries (2). In addition, a majority of older women were significantly single and less educated; they usually had lower-paying jobs, so they seldom had as much income to pay for medical care in the hospitals, similar to another study (3). The housewife position of women could explicate their low physical activity outdoors and their financial dependency correlated to their children or relatives (74.87%). These findings are consistent with previous studies (2). On another hand, at least 87% of elderly people suffered from many chronic conditions. The musculoskeletal (38.7%), cardiovascular (37.7%), and gastrointestinal disorders (24.6%) were significantly the most common morbidities appeared among older women in this study (*P*<0.05). Similar findings were reported elsewhere (4). A relationship between musculoskeletal diseases and negative determinants of health, such as overweight, low education level, poor health status and sedentary lifestyle in elderly women (5).

**Table 1:** Socio-demographic characteristics and chronic morbidities elders, distributed by gender

***Variables and modalities***	***Elderly men (%)***	***Elderly women (%)***	**P-*value***
Age(yr)			
60 – 69	78 (46.2)	117 (58.8)	0.015
>=70	91 (53.8)	82 (41.2)	
Marital status			
Without partner	71 (42.0)	133 (66.8)	0.001
With partner	98 (58.0)	66 (33.2)	
Education level			
Illiterate	115 (68.0)	157 (78.9)	0.027
Primary school and above	34 (32.0)	42 (21.1)	
Previous occupation			
Low-income (1)	68 (40.2)	159 (79.9)	0.001
Middle and high-income (2)	101 (59.7)	40 (20.1)	
Number of children			
1 or more	111 (65.7)	149 (74.87)	0.040
0	58 (34.3)	50 (25.12)	
Reported co-morbidities			
With 1 or more illness	147 (87.0)	182 (91.5)	0.611
Heart diseases and hypertension	49 (29.0)	75 (37.7)	0.049
Respiratory diseases and asthma	9 (5.3)	11 (5.5)	0.932
Infectious diseases	6 (3.6)	1 (0.5)	0.033
Skin diseases	5 (3.0)	3 (1.5)	0.341
Musculoskeletal diseases and arthritis	33 (19.5)	77 (38.7)	0.001
Gastrointestinal diseases	18 (10.7)	49 (24.6)	0.001
Diabetes and metabolic disorders	36 (21.3)	42 (21.1)	0.963
Urology and kidney diseases	17 (10.1)	11 (5.5)	0.102
Visual disorders	56 (33.1)	63 (31.7)	0.763

The higher proportion of gastrointestinal disorders was seen in older women (24.6%). Women were more likely to complain about irritable bowel, constipation, bloating, swollen belly, tight clothing and asthenia (6). Women are the vulnerable group to chronic diseases. Improving their socio-economic levels and health status require more attention on the part of politicians, civil society, geriatricians and community nurses.
